# Role of Steroid Therapy in Preventing Recurrence of Atrial Fibrillation After Ablation: A Systematic Review and Meta-Analysis

**DOI:** 10.7759/cureus.72437

**Published:** 2024-10-26

**Authors:** Nava R Sharma, Saral Lamichhane, Sudarshan Gautam, Madalasa Pokhrel, Marlon E Rivera Boadla, Sajog Kansakar, Arjun Basnet, Prabal KC, Prakriti Lamichhane, Gregory Cunn

**Affiliations:** 1 Internal Medicine, Maimonides Medical Center, Brooklyn, USA; 2 Internal Medicine, NYCHH (New York City Health and Hospitals) Woodhull, Brooklyn, USA; 3 Cardiology, Maimonides Medical Center, Brooklyn, USA; 4 Infectious Disease, Maimonides Medical Center, Brooklyn, USA; 5 Pathology, Kist Medical College, Lalitpur, NPL

**Keywords:** af recurrence, atrial fibrillation ablation, atrial fibrillation management, persistent atrial fibrillation, pulse corticosteroid therapy

## Abstract

Atrial fibrillation (AF) is the most common cardiac arrhythmia with challenging management due to its potential complications and high recurrence rates. Catheter ablation is a standard treatment option for symptomatic patients, particularly those unresponsive to medical management but has variable success rates. Inflammation plays a critical role in the pathogenesis and persistence of AF. This systematic review aims to study the potential benefits of corticosteroid use in the prevention of AF recurrence after procedures such as catheter ablation, providing a more comprehensive understanding of their role in improving outcomes. Four randomized controlled trials (RCTs) with 824 total participants and four cohort studies with 1128 participants were included for qualitative and quantitative analysis. All RCTs and cohort studies suggest no significant benefit of corticosteroids with ablation in preventing short-term (less than three months) AF recurrence. However, RCTs indicate that corticosteroid use with ablation significantly reduces the recurrence of AF over three months, but such a statistically significant effect is not seen in cohort studies for AF recurrence up to one year. This suggests that there might be some beneficial role of using steroids with ablation procedures in preventing recurrences of AF, but further large-scale studies are warranted for better evidence to support the use of steroids with ablation. Future research should focus on understanding the optimal duration and dosing of corticosteroid treatment to maximize benefits and minimize risks, especially in the immediate post-treatment period where the data currently show less clarity and higher variability. Additionally, further studies should explore the mechanisms through which corticosteroids exert their effects over different durations to better tailor treatment protocols.

## Introduction and background

Atrial fibrillation (AF) is the most common cardiac arrhythmia, significantly increasing the risk of stroke and heart failure [1]. The prevalence of AF rises with age, and its management remains a clinical challenge due to its potential complications and high recurrence rates [2]. Catheter ablation has emerged as a standard treatment option for patients with symptomatic AF, particularly those unresponsive to medical management [3]. Despite its efficacy, the success of ablation varies, with many patients experiencing recurrence of AF, necessitating further exploration of contributing factors and treatment options. Recovery rates after catheter ablation demonstrate considerable variation. Studies indicate that the overall recovery rate is around 53.1%, with paroxysmal AF showing a slightly higher success rate at 54.1%, while persistent AF patients exhibit a lower recovery rate at 41.8% [3,4]. These disparities highlight the complexity of AF as a condition and the need for personalized approaches to treatment and post-procedure care.

Evidence suggests that inflammation plays a critical role in the pathophysiology and persistence of AF [5,6]. Elevated inflammatory marker levels, such as C-reactive protein (CRP) and interleukin-6 (IL-6), are associated with the development and recurrence of AF [6]. This has led to increasing interest in targeting inflammation as a potential therapeutic approach to managing AF, particularly in reducing the risk of recurrence after ablation [5-7]. Corticosteroids, known for their potent anti-inflammatory properties, have been explored for their potential role in preventing AF recurrence [8]. While corticosteroids effectively reduce inflammation, their specific impact on AF recurrence post-ablation remains under-investigated [9]. A systematic review of available evidence is needed to clarify the potential benefits and risks of corticosteroid use in AF management after catheter ablation, providing a more comprehensive understanding of their role in improving outcomes.

## Review

Methodology

This systematic review was conducted in accordance with PROSPERO registration guidelines (ID: CRD42023415829). To identify relevant studies published up to May 2024, a comprehensive literature search was performed across PubMed, Cochrane Library, Embase, and ClinicalTrials.gov. The search strategy employed Medical Subject Headings (MeSH) and free-text terms to capture all pertinent studies. Key search terms included “Atrial Fibrillation” OR “AF” OR “AFib” OR “Afib” in combination with “Glucocorticoids” OR “Steroid Therapy” OR “Steroid*” OR “Hydrocortisone” OR “Prednisone” OR “Prednisolone” OR “Corticosteroid*” OR “Hormone Therapy” OR “Methylprednisolone” OR “Dexamethasone.” For procedural terms, we utilized “Radiofrequency Ablation” OR “Pulmonary Vein Ablation” OR “Catheter Ablation” OR “Pulmonary Vein Isolation.”

**Figure 1:**
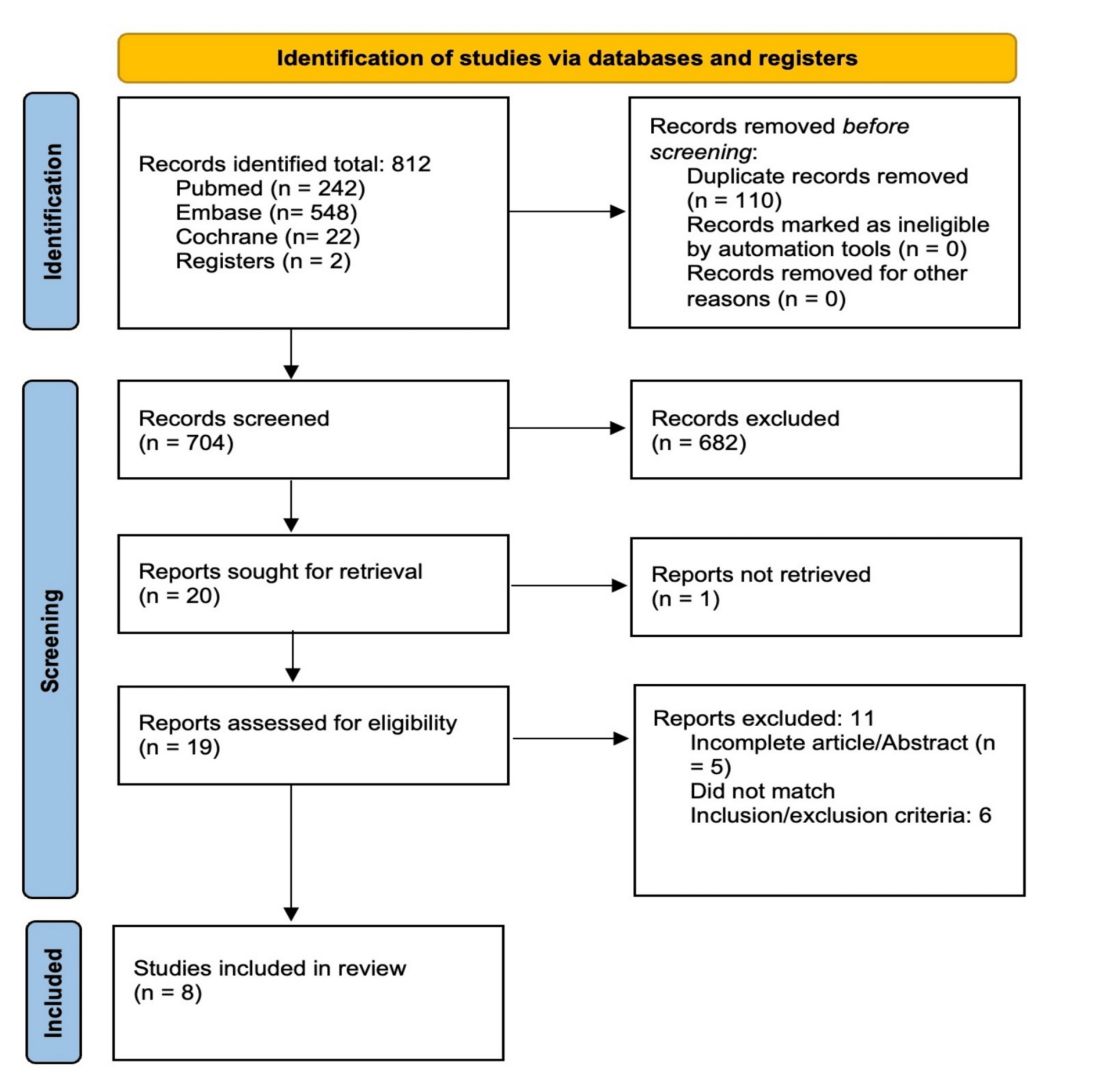
PRISMA flow diagram. Source: [10].

Four RCTs (n=824) and four cohort studies (n=1128) were included for qualitative and quantitative analysis. A PRISMA flow diagram was utilized to outline the study selection process as shown in Figure 1 [10].

Statistical analysis

The statistical analysis was conducted using RevMan version 5.4.1 (The Cochrane Collaboration, London, UK), focusing exclusively on dichotomous outcomes [11]. Odds ratios (ORs) were calculated for all outcomes. Using the DerSimonian and Laird method, a random-effects model was applied to account for potential heterogeneity across studies. Heterogeneity was assessed with the I² statistic, with values above 50% indicating substantial heterogeneity. The analysis included four RCTs and four cohort studies. Subgroup analyses were performed to explore variations across different study designs or follow-up periods. All statistical tests were two-sided, with a p-value <0.05 considered statistically significant.

Study selection: Inclusion and exclusion criteria

The inclusion criteria for this systematic review focused on studies involving human subjects aged 18 years and older with a confirmed diagnosis of AF of any subtype, including paroxysmal, persistent, or permanent AF. Eligible studies were required to investigate patients undergoing ablation procedures, such as radiofrequency (RF) ablation, pulmonary vein isolation (PVI), catheter ablation, or cryoablation along with the use of corticosteroids. Only studies that reported outcomes related to the recurrence or return of AF following these procedures were included in the review. Duration, dose, or type of steroid use was not taken into account.

Studies were excluded if they involved non-human subjects or utilized designs other than RCTs or cohort studies. Additionally, we excluded studies where patients were on long-term steroid therapy before undergoing the ablation procedure, as this could confound the evaluation of the post-procedural effects of corticosteroids on AF recurrence. By applying these criteria, we ensured the inclusion of high-quality evidence relevant to our research question.

Baseline characteristics

Basic Characteristics

The baseline characteristics of the included studies are summarized in Table 1.

**Table 1:** Basic characteristics of the studies N/A: not available; L: low-dose steroid; M: moderate-dose steroid; NS: non-significant.

Author, year	Age (years)			Total population		Male, n (%)			Female, n (%)			Paroxysmal AF, n (%)			Duration AF, months		
	Treatment	Control	p-Value	Treatment	Control	Treatment	Control	p-Value	Treatment	Control	p-Value	Treatment	Control	p-Value	Treatment	Control	p-Value
Andrade et al., 2013 [12]	57.4 ± 10.5	59.5 ± 8.3	NS	45	45	33 (73)	31 (69)	N/A	12 (27)	14 (31)	NS	45 (100)	45 (100)		48	48	NS
Won et al., 2013 [13]	55 ± 11	55 ± 11	0.78	89	120	70 (78)	94 (78)	0.96	19 (22)	26 (22)	N/A	50 (56)	57 (48)	0.26	N/A	N/A	N/A
Kim et al., 2015 [14]	L56 ± 9 M56 ±10	56 ± 10	0.93	L95, M97	95	L80 (84), M82 (84)	80 (85)	0.98	L15, M15	15	N/A	L58 (63), M61 (64)	60 (63)	N/A	N/A	N/A	N/A
Agboola et al., 2023 [15]	61.5 ± 8.2	62.2 ± 11.2	0.78	31	53	26 (83.9)	41 (77.4)	0.58	5 (16.1)	12 (22.6)	N/A	N/A	N/A	N/A	N/A	N/A	N/A
Koyama et al., 2010 [16]	59.8 ± 8.7	61.5 ± 10.3	0.33	60	65	48 (80)	52 (80)	1.0	12 (20)	13 (20)	N/A	60 (100)	65 (100)	N/A	8.8 ± 6.4	5.8 ± 4.6	<0.01
Kim et al., 2015 [17]	56 ± 10	56 ± 9	N/A	64	74	51 (79.7)	50 (67.6)		13 (20.3)	24 (32.4)	N/A	48 (75)	55 (74.3)	N/A	52	46	N/A
Iskandar et al., 2017 [18]	63 ± 8.7	63 ± 8.9	0.65	30	30	24 (80)	22 (73)	0.76	6 (20)	8 (27)		30 (100)	30 (100)	N/A	4.1 ± 4.1 yrs	6.9 ± 6.7 yrs	0.059

The age of participants across the studies was relatively consistent, with mean ages ranging from 55 to 63 years in both the treatment (T) and control (C) groups. The gender distribution showed a predominance of male participants, comprising 70-84% of the total population in most studies. This gender imbalance was consistent across both the treatment and control arms. The percentage of patients with paroxysmal AF varied widely, ranging from 45% to 100% in the treatment groups and from 48% to 100% in the control groups. The mean duration of AF was reported in only a few studies, with some demonstrating a significant difference between the treatment and control groups (e.g., Koyama et al. reported a mean AF duration of 8.8 ± 6.4 months in the treatment group vs. 5.8 ± 4.6 months in the control group, p < 0.01) [16]. Other studies did not find significant differences in AF duration (e.g., Iskandar et al.) [18]. Overall, the baseline characteristics of the treatment and control groups in these studies were generally well-balanced, suggesting that randomization effectively minimized confounding variables between groups. However, some variation in the duration and type of AF may influence the interpretation of treatment effects. It is also worth noting that the RCT by Melby D.P. does not provide the relevant baseline characteristics [19].

Comorbidities

The baseline comorbidities of the included studies are summarized in Table 2. The table presents the prevalence of various comorbidities in patients across different studies, comparing treatment and control groups. For congestive heart failure, the studies by Won H and Kim DR, et al. show no statistically significant differences between the treatment and control groups, with p-values of 0.67 and 0.44, respectively [13,14]. For hypertension, Andrade et al. [12] and Koyama et al. [16] observed no significant differences (p-values not significant). At the same time, Won [13] and Agboola et al. [15] reported similar findings (p-values of 0.89 and 0.36, respectively). For diabetes, only Won H found a statistically significant difference (p = 0.04), indicating a higher prevalence in the control group [13]. Other studies, such as Kim et al. [14] and Agboola et al. [15], reported no significant differences for diabetes, stroke, or coronary artery disease. This suggests no significant differences in the prevalence of these comorbidities between the treatment and control groups across the included studies, except for a possibly higher prevalence of diabetes in one study's control group.

**Table 2:** Baseline comorbidities of patients included in the studies L: low-dose steroid; M: moderate-dose steroid; NS: non-significant; N/A: not available.

Author, year	Congestive heart failure (n, %)			Hypertension (n, %)			Diabetes (n, %)			Stroke (n, %)			Coronary artery disease (n, %)			Congestive heart failure (n, %)		
	Treatment	Control	p-Value	Treatment	Control	p-Value	Treatment	Control	p-Value	Treatment	Control	p-Value	Treatment	Control	p-Value	Treatment	Control	p-Value
Andrade et al., 2013 [12]	N/A	N/A	N/A	11 (24)	16 (36)	NS	0 (0)	3 (7)	NS	N/A	N/A	N/A	N/A	N/A	N/A	N/A	N/A	N/A
Won et al., 2013 [13]	2 (2)	4 (3)	0.67	38 (45)	52 (44)	0.89	4 (5)	16 (14)	0.04	8 (9)	6 (5)	0.22	4 (5)	10 (8)	0.46	2 (2)	4 (3)	0.67
Kim et al., 2015 [14]	L3(3), M5(5)	2 (2)	0.44	L46 (48), M49(52)	40 (42)	0.38	L6 (6), M13 (14)	10 (10)	0.24	L6, M9	3	0.19	L1, M3	2	0.54	L3 (3), M5 (5)	2 (2)	0.44
Agboola et al., 2021 [15]	4 (12.9)	17 (32.1)	0.07	17 (54.8)	35 (66.0)	0.36	5 (16.1)	5 (9.4)	0.49	2 (6.5)	7(13.2)	0.47	4 (12.9)	11 (20.8)	0.56	4 (12.9)	11 (20.8)	0.56
Koyama et al., 2010 [16]	N/A	N/A	N/A	36 (55.4)	29 (44.6)	0.87	N/A	N/A	N/A	N/A	N/A	N/A	4 (6.7)	6 (9.2)	0.60	N/A	N/A	N/A
Kim et al., 2015 [17]	8 (12.5)	3 (4.1)	N/A	21 (32.8)	27 (36.5)	N/A	8 (12.5)	10 (13.5)	N/A	6 (9.4)	4 (5.4)	N/A	N/A	N/A	N/A	8 (12.5)	3 (4.1)	N/A
Iskandar et al., 2017 [18]	N/A	N/A	N/A	18 (60)	12 (40)	0.19	N/A	N/A		N/A	N/A	N/A	12 (40)	9 (30)	0.58	N/A	N/A	N/A

Procedural Characteristics

The baseline procedural characteristics of the included studies are summarized in Table 3. The table provides an overview of procedural characteristics and ablation techniques used across multiple studies, comparing treatment and control groups. The parameters include circumferential PVI, cavotricuspid isthmus (CTI) ablation, linear ablation, complex fractionated atrial electrogram (CFAE) ablation, superior vena cava (SVC) isolation, total procedure duration, fluoroscopy time, and RF ablation time. For circumferential PV isolation, all patients in Won [13], Agboola et al. [15], and Kim et al. [17] underwent the procedure in both treatment and control groups (100%). The rates of CTI ablation were similar between groups, with no statistically significant differences reported. For linear ablation, studies like Won [13] and Kim et al. [14] showed comparable prevalence between groups, with no significant differences. CFAE ablation was uncommon, and SVC isolation was performed at low rates, as seen in Won et al. [13].

**Table 3:** Procedural characteristics PV: pulmonary vein; CTI: cavotricuspid isthmus; CFAE: complex fractionated atrial electrogram; SVC: superior vena cava; RF: radiofrequency; N/A: not available; L: low-dose steroid; M: moderate-dose steroid; NS: non-significant.

Author, year	Circumferential PV isolation (n, %)			CTI ablation (n, %)			Linear ablation (n, %)			CFAE (n,%)			SVC isolation (n, %)			Total duration of the procedure (min)			Total fluoroscopy time (min)			Duration of RF ablation (min)		
	Treatment	Control	p-Value	Treatment	Control	p-Value	Treatment	Control	p-Value	Treatment	Control	p-Value	Treatment	Control	p-Value	Treatment	Control	p-Value	Treatment	Control	p-Value	Treatment	Control	p-Value
Andrade et al., 2013 [12]	N/A	N/A	N/A	N/A	N/A	N/A	N/A	N/A	N/A	N/A	N/A	N/A	N/A	N/A	N/A	169 ± 35	182 ± 60	NS	35 ± 8	42 ± 20	NS	58 ± 21	48 ± 19	0.006
Won et al., 2013 [13]	89 (100)	120 (100)	0.99	86 (97)	112 (93)	0.36	37 (42)	61 (51)	N/A	0 (0)	1 (1)	0.39	4 (5)	7 (6)	0.76	188 ± 45	201 ± 52	0.06	47 ± 21	48 ± 16	0.50	82 ± 28	89 ± 32	0.01
Kim et al., 2015 [14]	L95, M97	95	0.37	L88, M84	81	0.09	L36, M35	35	0.95	L2, M7	7	0.19	L2 (2), M14 (15)	6 (7)	0.01	L182 ± 46, M168 ± 40	192 ± 52	0.13	L58 ± 19, M43 ± 13	48 ± 16	0.68	L77 ± 27, M68 ± 23	86 ± 24	0.98
Agboola et al., 2021 [15]	31 (100)	53 (100)	N/A	14 (45.2)	19 (35.8)	N/A	12 (38.7)	16 (30.2)	N/A	N/A	N/A	N/A	N/A	N/A	N/A	N/A	N/A	N/A	N/A	N/A	N/A	N/A	N/A	N/A
Koyama et al., 2010 [16]	N/A	N/A	N/A	N/A	N/A	N/A	N/A	N/A	N/A	N/A	N/A	N/A	4 (6.6)	3 (4.6)	0.62	200.8 ± 35.6	209.1 ± 32.6	0.27	81.4 ± 22.3	82.6 ± 24.3	0.42	47.9 ±15.8	48.2 ± 19.3	0.94
Kim et al., 2015 [17]	64 (100)	74 (100)	N/A	47 (73.4)	56 (75.7)	N/A	N/A	N/A	N/A	N/A	N/A	N/A	6 (9.4)	8 (10.8)	N/A	363 ± 94	390 ± 99	N/A	37 ± 9	38 ± 8	N/A	118 ± 38	127 ± 38	N/A
Iskandar et al., 2017 [18]	N/A	N/A	N/A	N/A	N/A	N/A	N/A	N/A	N/A	N/A	N/A	N/A	N/A	N/A	N/A	174 ± 38	166 ± 47	0.52	54.7 ± 16.5	54.8 ± 17.2	0.9	56 ± 10.7	51 ± 13.5	0.11

The total procedure duration varied across studies. Andrade et al. [12] and Kim et al. [14] reported no significant difference between groups. However, Andrade et al. [12] found a significantly higher RF ablation duration in the treatment group (p = 0.006), while Won [13] observed higher RF ablation time in the control group (p = 0.01). Fluoroscopy time remained consistent across studies, indicating similar radiation exposure regardless of the intervention type. The table suggests that while procedural strategies and ablation times varied slightly between groups, general procedural parameters were broadly similar across most studies, with few statistically significant differences observed.

Inflammatory Markers

The baseline inflammatory markers of the included studies are summarized in Table 4. This table compares key clinical variables such as WBC, CRP, maximal body temperature, and the incidence of pericarditis across several studies included in a systematic review. Some studies, including those by Andrade et al. [12], Agboola et al. [15], and Iskandar et al. [18], did not report these parameters. However, data from Won H. reveal no statistically significant differences between treatment and control groups for WBC (p = 0.50), CRP levels (p = 0.91), body temperature (p = 0.27), or the incidence of pericarditis (1% vs. 3%, p = 0.47) [13]. Similarly, Kim DR et al. compared treatment subgroups and found no significant differences in WBC or the incidence of pericarditis. Still, CRP levels were significantly lower in the treatment subgroups (p = 0.01) [14]. Kim YR et al. reported a considerably lower WBC count (p = 0.03) and maximal body temperature (p < 0.001) in the treatment group compared to the control group [17]. These findings suggest varying degrees of inflammatory response across the included studies, with some demonstrating significant intergroup differences and others showing comparable outcomes.

**Table 4:** Inflammatory markers N/A: not available; L: low-dose steroid; M: moderate-dose steroid; WBC: white blood cell; CRP: C- reactive protein.

Author, year	WBC (/μL)			CRP (mg/L)			Maximal body temperature (°C)			Pericarditis (%)		
	Treatment	Control	p-Value	Treatment	Control	p-Value	Treatment	Control	p-Value	Treatment	Control	p-Value
Andrade et al., 2013 [12]	N/A	N/A	N/A	N/A	N/A	N/A	N/A	N/A	N/A	N/A	N/A	N/A
Won et al., 2013 [13]	8796 ± 2279	9078 ± 2523	0.50	22.2 ± 26.8	20.6 ± 11.9	0.91	37.0 ± 0.5	37.1 ± 0.4	0.27	1 (1%)	3 (3%)	0.47
Kim et al., 2015 [14]	L8880 ± 2332, M9122 ± 2559	9279 ± 2668	0.61	L20.3 ± 12.8, M5.2 ± 8.4	23.7 ± 13.4	0.01	L37.0 ± 0.5, M36.8 ± 0.2	37.1 ± 0.5	0.01	L2 (2.1), M0 (0)	1 (1.1)	0.52
Agboola et al., 2021 [15]	N/A	N/A	N/A	N/A	N/A	N/A	N/A	N/A	N/A	N/A	N/A	N/A
Koyama et al., 2010 [16]	N/A	N/A	N/A	N/A	N/A	N/A	N/A	N/A	N/A	N/A	N/A	N/A
Kim et al., 2015 [17]	6782 ± 1895	9840 ± 2531	0.03	N/A	N/A	N/A	36.9 ± 0.3	37.1 ± 0.3	<0.001	N/A	N/A	N/A
Iskandar et al., 2017 [18]	N/A	N/A	N/A	N/A	N/A	N/A	N/A	N/A	N/A	N/A	N/A	N/A

Recurrence of AF Outcome

Table 5 provides an overview of AF recurrence rates across multiple time points, as reported in various studies. These studies employed different designs, including cohort studies, retrospective cohort studies, and RCTs, with varying follow-up durations and recurrence outcome measures.

**Table 5:** Recurrence of atrial fibrillation outcome table N/A: not available; T: treatment; C: control; L: low-dose steroid; M: moderate-dose steroid.

Author, year	Study design	Less than 3 days			3 to 30 days (n, %)			Less than 3 months			3-12 months			12 months			1-14 months			3-24 months		
		Treatment	Control	p-Value	Treatment	Control	p-Value	Treatment	Control	P-Value	Treatment	Control	p-Value	Treatment	Control	p-Value	Treatment	Control	p-Value	Treatment	Control	p-Value
Andrade et al., 2013 [12]	Cohort study	N/A	N/A	N/A	N/A	N/A	N/A	48.9%	51.1%	N/A	N/A	N/A	N/A	28.9%	37.8%	N/A	N/A	N/A	N/A	N/A	N/A	N/A
Won et al., 2013 [13]	Cohort study	11/89 (12%)	17/117 (15%)	N/A	11/89 (12%)	13/117 (11%)	N/A	N/A	N/A	N/A	N/A	N/A	N/A	13/89 (15%)	26/117 (22%)	N/A	N/A	N/A	N/A	N/A	N/A	N/A
Kim et al., 2015 [14]	Cohort study	N/A	N/A	N/A	N/A	N/A	N/A	L24 (25%), M25 (26%)	24 (25%)	0.99	L23 (24%), M18 (19%)	22 (23%)	0.12	N/A	N/A	N/A	N/A	N/A	N/A	N/A	N/A	N/A
Agboola et al., 2021 [15]	Retrospective cohort	N/A	N/A	N/A	N/A	N/A	N/A	24 (25%)	N/A	N/A	N/A	N/A	N/A	N/A	N/A	N/A	N/A	N/A	N/A	N/A	N/A	N/A
Koyama et al., 2010 [16]	Prospective, randomized, double-blind study	4 (7)	20 (31)	<0.001	12 (20)	12 (18)	0.9	N/A	N/A	N/A	N/A	N/A	N/A	N/A	N/A	N/A	9 (15)	19(29)	N/A	N/A	N/A	N/A
Kim et al., 2015 [17]	Prospective, randomized, single-blind study	5 (7.8)	9 (12.2)	N/A	11 (17.2)	20 (27.0)	N/A	15 (23.4)	36 (48.6)	0.003	N/A	N/A	N/A	N/A	N/A	N/A	N/A	N/A	N/A	23 (35.9)	25 (33.8)	0.858
Iskandar et al., 2017 [18]	Prospective, randomized, double-blinded study	N/A	N/A	N/A	N/A	N/A	N/A	8 (27)	5 (17)	0.347	12 (40)	9 (30)	0.41	N/A	N/A	N/A	N/A	N/A	N/A	N/A	N/A	N/A
Melby et al., 2019 [19]	Double-blinded clinical trial	N/A	N/A	N/A	N/A	N/A	N/A	N/A	N/A	N/A	4/58	12/58		N/A	N/A	N/A	N/A	N/A	N/A	N/A	N/A	N/A

Several studies focused on short-term recurrence within three to 30 days. For instance, Won [13] reported similar recurrence rates in the treatment (12%) and control groups (15%) within three days, while Koyama et al. [16] observed a significantly lower recurrence rate in the treatment group (7%) compared to the control group (31%, p < 0.001). In the three- to 30-day window, recurrence rates were more consistent between groups. For example, Kim YR et al. noted a recurrence of 17.2% in the treatment group versus 27.0% in the control group [17].

Longer-term recurrence outcomes were reported by Andrade et al. [12], Won [13], Kim et al. [17], and Melby [19]. Andrade et al. found that the control group had a higher recurrence at 12 months (37.8%) compared to the treatment group (28.9%) [12]. Kim et al. [17] reported a significant difference in recurrence within three months (23.4% vs. 48.6%, p = 0.003), while at three to 24 months, recurrence rates equalized between groups (35.9% vs. 33.8%, p = 0.858). Melby DP did not provide specific recurrence rates but indicated comparable long-term outcomes between treatment and control groups [19].

Results

Observational Studies

**Figure 2:**
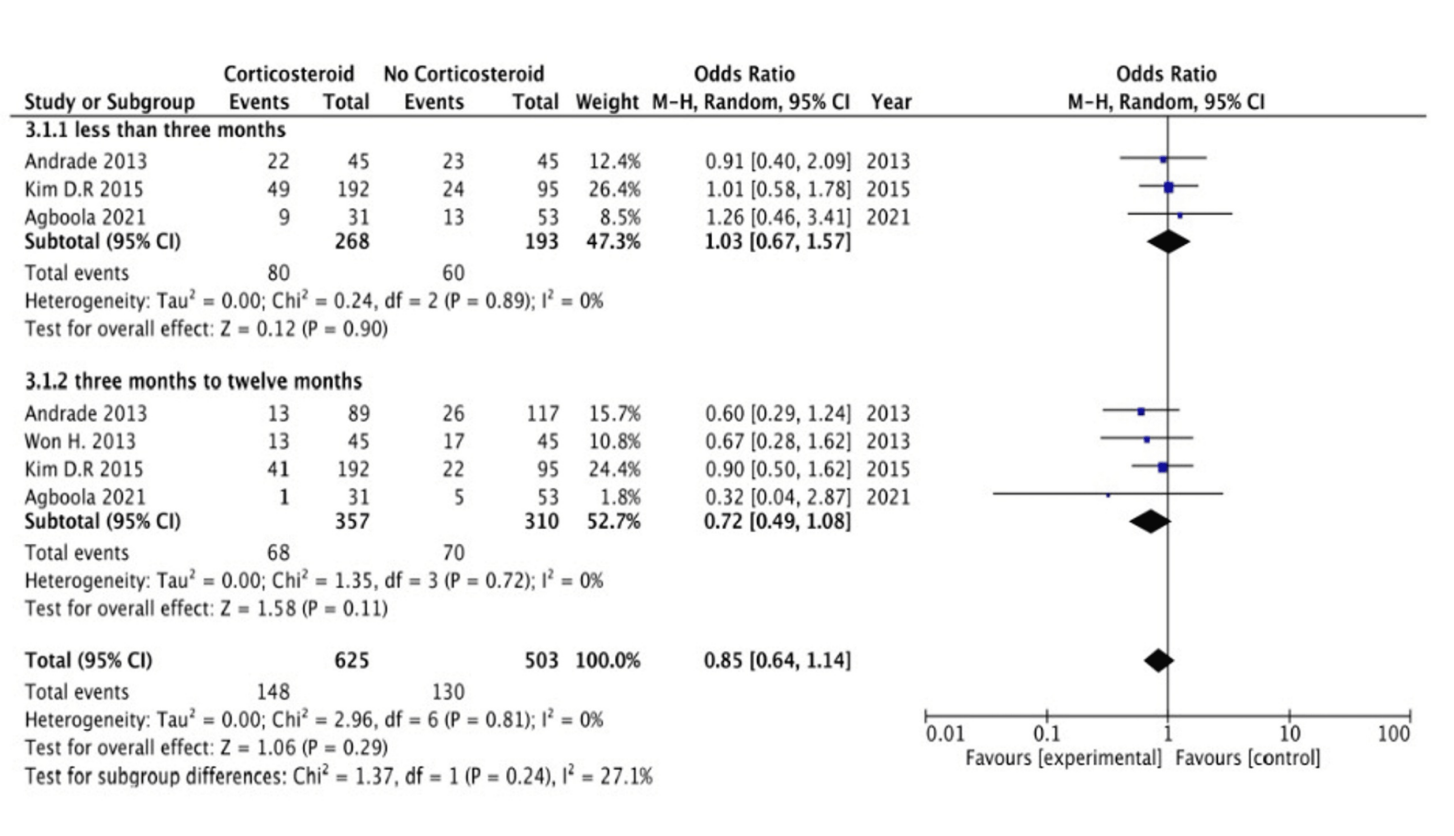
Forest plot showing outcomes from observational studies References: [12-15].

Figure 2 presents a forest plot illustrating the recurrence of AF in various observational studies.

AF recurrence less than three months: The subgroup analysis evaluating AF recurrence within the first three months with corticosteroid use with ablation showed no statistically significant impact on recurrence rates. The pooled OR was 1.03 (95% CI: 0.67 to 1.57), indicating that the likelihood of AF recurrence was similar in both the corticosteroid and control groups. The confidence interval crossing 1 suggests that corticosteroid use does not provide a clear benefit or harm in preventing AF recurrence during this early period. Moreover, this subgroup's low heterogeneity (I² = 0%) suggests consistent findings across the included studies, reinforcing that corticosteroid administration does not significantly influence the risk of early AF recurrence within the initial three months.

AF recurrence from three to 12 months: In this subgroup, corticosteroid use with ablation did not significantly impact recurrence rates. The pooled OR was 0.72 (95% CI: 0.49 to 1.08), suggesting a trend toward a lower risk of AF recurrence with corticosteroid use, but the effect was not statistically significant. This result indicates that corticosteroid use does not significantly reduce long-term AF recurrence risk. The low heterogeneity (I² = 0%) within this subgroup shows consistency among the studies, suggesting that corticosteroid use does not play a decisive role in modifying AF recurrence rates for a short term or a longer term within the first year.

Randomized Controlled Trials

**Figure 3:**
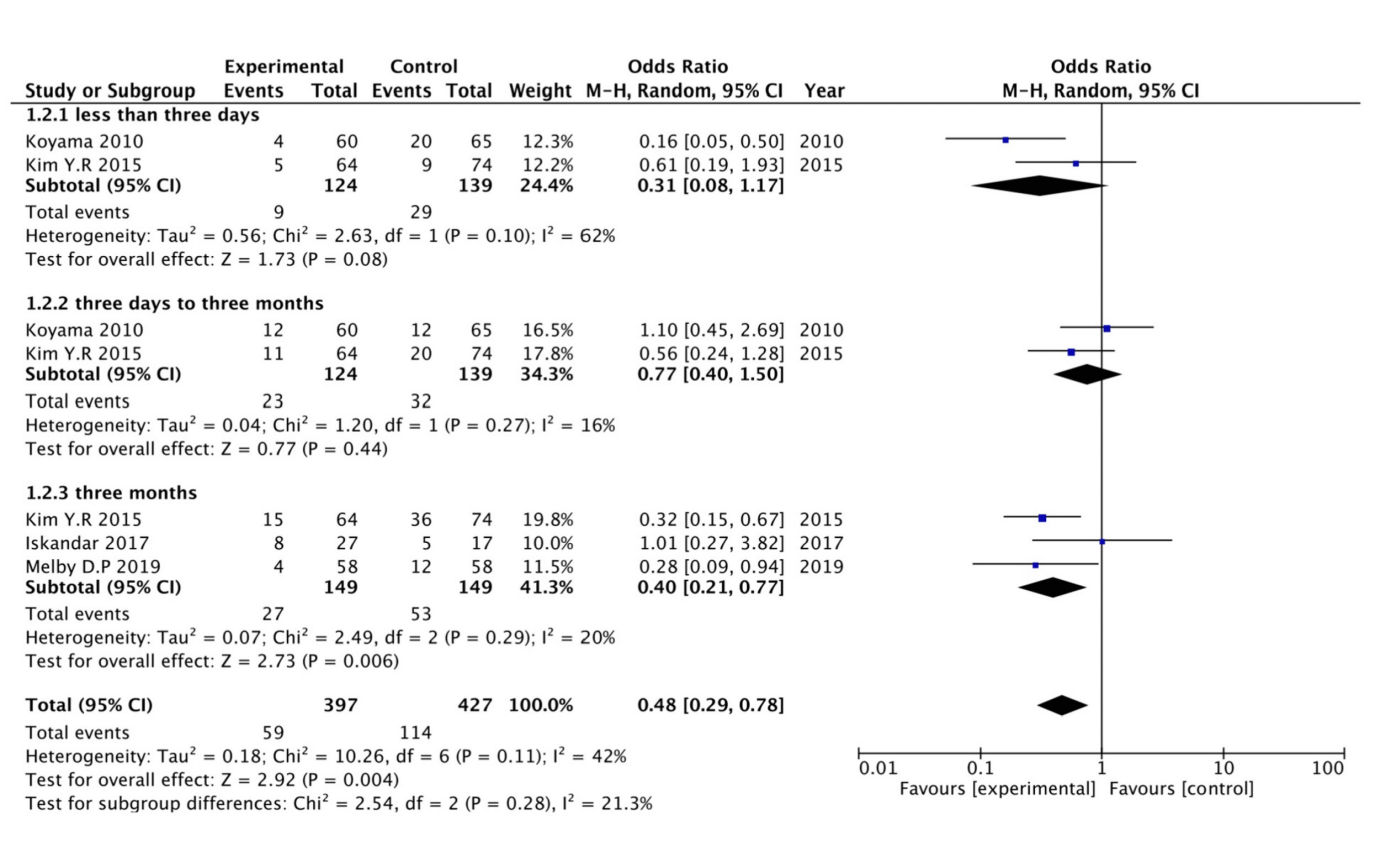
Forest plot showing outcomes from RCTs. References: [16-19]. RCTs, randomized controlled trials.

Figure 3 presents a forest plot illustrating the recurrence of AF in various RCTs.

AF recurrence less than three days: The subgroup analysis of RCTs assessing AF recurrence in less than three days with corticosteroid use with ablation (subgroup 1.1.1) included two studies: Koyama 2010 and Kim 2015 [16,17]. The pooled OR for this subgroup was 0.31 (95% CI: 0.08 to 1.17), suggesting a trend toward a reduced risk of AF recurrence during this very early period with corticosteroid use. Although the results are not statistically significant (confidence interval crosses 1), the trend may indicate a potential early benefit of corticosteroids. However, there was moderate heterogeneity within this subgroup (I² = 62%), which suggests some variability in the outcomes of the included studies. Further studies may be needed to confirm if corticosteroid use effectively reduces AF recurrence risk within the first three days.

AF recurrence from three to 30 days: The analysis of AF recurrence from three to 30 days with corticosteroid use with ablation (subgroup 1.1.2) also included two studies, Koyama 2010 and Kim 2015 [16,17]. The pooled OR for this subgroup was 0.77 (95% CI: 0.40 to 1.50), which suggests no statistically significant difference in AF recurrence from three to 30 days between the corticosteroid and no corticosteroid groups. The confidence interval crossing 1 indicates that corticosteroid administration does not impact AF recurrence risk within three to 30 days. Additionally, the heterogeneity within this subgroup was low (I² = 16%), suggesting that the results are consistent across the studies.

AF recurrence within three months: The subgroup examining AF recurrence over three months with corticosteroid use with ablation (subgroup 1.1.3) included three studies: Kim 2015, Iskandar 2017, and Melby 2019 [17-19]. The pooled OR for this subgroup was 0.40 (95% CI: 0.21 to 0.77), showing a statistically significant reduction in AF recurrence over three months with corticosteroid use. This result suggests that corticosteroid administration with ablation may effectively prevent AF recurrence in three months. The low heterogeneity (I² = 20%) within this subgroup indicates that the results are consistent across studies, supporting the robustness of this finding. The statistically significant reduction in AF recurrence in this subgroup highlights the potential benefit of using corticosteroids with ablation procedures for preventing AF recurrence.

Risk-of-Bias Assessment of the Studies

**Figure 4:**
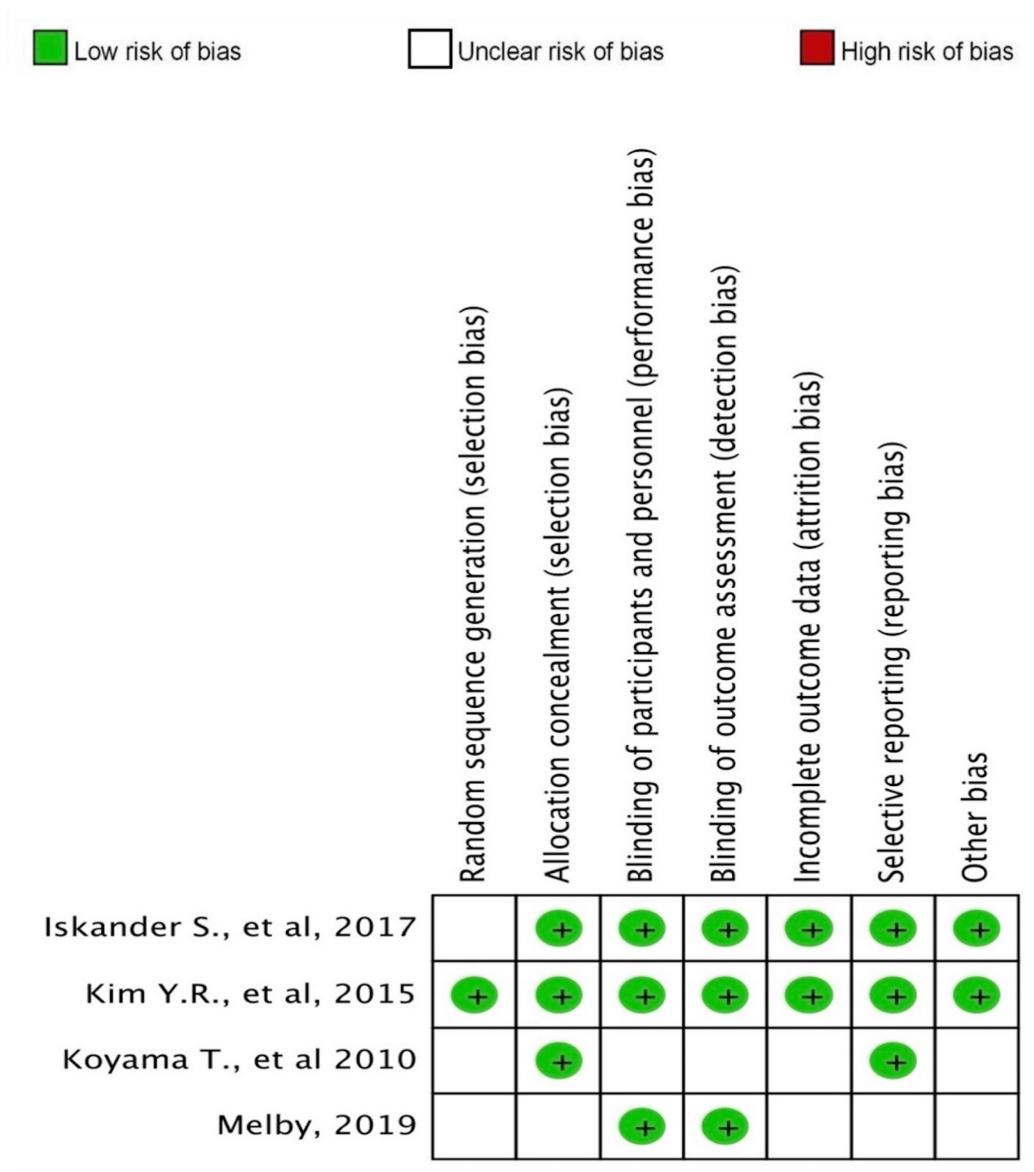
Risk-of-bias assessment of randomized controlled trials References: [16-19].

The risk-of-bias evaluation for both cohort studies and RCTs is summarized in Table 6 and illustrated in Figure 4. Overall, the assessment revealed a low risk of bias across most studies. This indicates that the methodologies employed in these studies were robust, with minimal concerns regarding the validity of their findings.

**Table 6:** Risk-of-bias assessment of cohort studies

Study	Representativeness of the exposed cohort	Selection of the non-exposed cohort	Ascertainment of exposure	Demonstration that outcome of interest was not present at the start of the study	Comparability of cohorts on the basis of the design or analysis	Assessment of outcome	Follow-up long enough for outcomes to occur	Adequacy of follow-up of cohort	Total score
Kim et al., 2015 [14]	1	1	1	1	2	1	1	1	9
Won et al., 2013 [13]	1	1	1	1	2	1	1	1	9
Andrade et al., 2013 [12]	1	1	1	1	1	1	1	1	8
Agboola et al., 2021 [15]	1	1	1	1	1	1	1	1	8

Discussion

AF is the most frequently encountered arrhythmia in clinical settings and is linked to an increased risk of mortality, stroke, and peripheral embolism [1,9]. Based on data from the Global Burden of Disease study, AF is estimated to affect up to 33.5 million people globally [20]. Furthermore, its prevalence is projected to double over the next four decades [20,21]. Despite significant research efforts to better understand its pathophysiology and develop more effective treatments, pinpointing the specific causative mechanisms in individual patients remains challenging, and the effectiveness of existing therapies remains suboptimal. As a result, the incidence of AF continues to rise, underscoring the need for innovative therapeutic approaches [21,22].

Risk Factors

AF is a progressive condition that often begins as nonsustained episodes triggered by ectopic activity and can advance to persistent AF as the atrial myocardial substrate undergoes pathological changes [23]. Research indicates that several risk factors contribute to the development of AF, including older age, male sex, and European ancestry. Additional modifiable factors, such as physical inactivity, smoking, obesity, diabetes mellitus, obstructive sleep apnea, and hypertension, have been shown to promote atrial structural and electrical remodeling, thereby increasing susceptibility to AF [24].

Etiopathogenesis of AF and Its Recurrence

Understanding the cellular, molecular, and electrophysiological changes leading to AF remains challenging due to its complex nature and the diverse pathophysiological mechanisms. These mechanisms can vary significantly among patients and species [21,24]. A crucial factor influencing atrial electrophysiology and structural changes is inflammation, which enhances the susceptibility to AF. Inflammatory processes can disrupt calcium homeostasis and affect connexin expression, both critical in the triggers of AF and the heterogeneous conduction within the atria [25]. Furthermore, inflammation leads to structural remodeling of the atrial tissue, characterized by myolysis, cardiomyocyte apoptosis, and the activation of fibrotic pathways involving fibroblasts, transforming growth factor-β, and matrix metalloproteinases. These alterations culminate in changes to atrial architecture and function [26,27].

In addition to direct electrophysiological effects, inflammation, endothelial dysfunction, and platelet activation contribute to a prothrombotic and proinflammatory state commonly associated with AF [28]. Biomarkers such as CRP and IL-6 play a role in promoting the production of tissue factor and von Willebrand factor (vWF), which worsen endothelial dysfunction and enhance coagulation while impairing fibrinolysis [29]. The interaction between vWF and its glycoprotein Ib receptor (vWF-GPIb) triggers "thrombo-inflammatory" pathways, further increasing the risk of thromboembolic events [27,29,30]. Platelet activation, often mediated by interactions with leukocytes or monocytes, significantly contributes to thrombosis in AF. Biochemical pathways, such as CD40-CD40 ligand and P-selectin-P-selectin glycoprotein ligand 1, intensify these interactions, reinforcing the prothrombotic environment in AF [31-33].

Key contributors to thrombus formation include abnormalities in endothelial function, platelet activation, coagulation, fibrinolysis, and inflammatory markers. Among the hemostatic markers, thrombin-antithrombin complex (TAT), plasminogen activator inhibitor-1 (PAI-1), and D-dimer are particularly noteworthy [31]. D-dimer, a product of fibrin degradation, serves as a well-established biomarker for assessing the activation of coagulation and fibrinolysis [34]. TAT levels provide insight into thrombin activity, while PAI-1, the principal inhibitor of tissue-type plasminogen activator (tPA), is critical for regulating the fibrinolytic pathway. Our meta-analysis indicates that elevated levels of TAT, PAI-1, and D- dimer are associated with an increased risk of stroke and thromboembolic events in patients with AF [35,36].

In AF pathogenesis, various biomarkers play significant roles in modulating the risk of stroke and thromboembolic events, primarily through their involvement in inflammation, coagulation, and endothelial function. While biomarkers like D-dimer, TAT, and PAI-1 exhibit strong associations with adverse outcomes in AF, others, such as CRP, prothrombin fragment, and P-selectin, have not consistently shown similar associations [37]. Some review studies have reported no significant correlations between these biomarkers and the risk of stroke or thromboembolic events in AF. This may stem from limited sample sizes and reduced statistical power in those analyses. Therefore, large-scale epidemiological studies are needed to investigate these relationships further and assess their clinical relevance [38].

Moreover, consistently elevated circulating levels of PAI-1 and TAT have been linked to an increased risk of stroke in AF patients. In contrast, elevated D-dimer levels are associated with a greater likelihood of subsequent thromboembolic events. Preliminary findings also suggest that other biomarkers, including IL-6, vWF, and mean platelet volume, may contribute to the pathogenesis of AF and its complications; however, more comprehensive research is required to establish their roles definitively [36].

Additionally, accumulating evidence indicates a significant link between oxidative stress and AF. In the atrial myocardium during AF, substantial oxidative damage may contribute to atrial remodeling [39]. Various pathophysiological changes associated with increased oxidative stress have been identified, including alterations in gene transcription profiles, mitochondrial DNA damage, heightened activity of enzymes such as NAD(P)H oxidase and xanthine oxidase, and activation of inflammatory processes and the renin-angiotensin system [40]. Oxidative stress also plays a role in several cardiovascular disorders and risk factors that predispose individuals to AF. Preliminary studies involving dietary antioxidants like vitamin C have shown promising results in mitigating oxidative stress [41]. Further insights have emerged from research on agents with pleiotropic effects, such as renin-angiotensin system inhibitors, statins, corticosteroids, and carvedilol, which possess antioxidant properties [42]. Continued research is essential to clarify the impact of oxidative stress on atrial remodeling, as understanding these processes in the context of AF could pave the way for innovative therapeutic strategies [43].

Treatment of AF Recurrence

Catheter ablation is frequently utilized for managing AF, particularly in patients who remain symptomatic despite antiarrhythmic drug therapy [44]. Its primary aim is to alleviate symptoms, enhance quality of life, and decrease the frequency of arrhythmia episodes, particularly in individuals with paroxysmal AF. In some instances, it may also be considered as a first-line option for paroxysmal AF patients without significant comorbid conditions [44,45]. The two main techniques for catheter ablation are RF ablation and cryoablation. RF ablation uses heat energy to create lesions in the atrial tissue, isolating the pulmonary veins (PVs), commonly the source of ectopic electrical activity that initiates AF. Cryoablation achieves similar results using extreme cold temperatures. PVI remains the cornerstone of AF ablation therapy, but additional areas may be targeted based on the patient’s arrhythmia characteristics and clinical presentation [46].

Catheter ablation has been shown to significantly reduce the recurrence of AF, with a 48% reduction in any AF recurrence and a 51% reduction in symptomatic AF when compared to drug therapy over a five-year follow-up period, as demonstrated in the Catheter Ablation vs. Antiarrhythmic Drug Therapy for Atrial Fibrillation (CABANA) trial [47]. The procedure also markedly decreases the overall burden of AF, regardless of the initial type of AF presented by the patient [47].

However, more than 30% of patients experience early recurrence of AF within the first three months following ablation, a period commonly referred to as the "blanking period" [17]. This early recurrence can be highly symptomatic and may require treatment through medications, cardioversion, or even hospitalization [17]. Steroid therapy has been investigated for its role in modulating the acute inflammatory response that occurs after ablation, as it can reduce tissue swelling and inflammatory cell infiltration around the ablation site. In one prospective study, administering a single intravenous dose of 250 mg hydrocortisone immediately after trans-septal puncture revealed dormant PV conduction during PVI [17]. Despite these acute effects, the study found no significant difference in long-term PV reconnections or freedom from AF recurrence at one year between patients who received steroids and those who did not [17]. Thus, while steroids may impact early post-ablation inflammation and PV activity, their long-term benefit in reducing AF recurrence remains uncertain.

Role of Steroid in AF Recurrence

Steroid therapy may also reduce delayed scar formation during the healing process. Steroids affect scar maturation by inhibiting the proliferation of fibroblasts and promoting their apoptosis [17,19]. This may, however, be a double-edged sword, although steroids may help reduce abnormal uneven fibrosis (an anatomic substrate for focal triggering or micro-reentry). They may also inhibit the delayed extension of the RF lesion between the ablation points and promote LR [17,18].

The meta-analysis of cohort studies compares outcomes between patients treated with ablation procedures with corticosteroids and those who were not; outcomes were categorized by the follow-up time intervals. For less than three months, the OR is 1.03 (95% CI: 0.67 to 1.57), indicating no statistically significant difference between the corticosteroid and control groups (p = 0.90). Between three and 12 months, the OR is 0.72 (95% CI: 0.49 to 1.08), showing a trend toward benefit with corticosteroid use, though the result is not statistically significant (p = 0.11). Overall, when data from both periods are combined, the total OR is 0.85 (95% CI: 0.64 to 1.14), again demonstrating no significant difference between the groups (p = 0.29). There is no significant heterogeneity between subgroups (p = 0.24), indicating consistent findings across different durations of corticosteroid use. Thus, the cohort study suggests that corticosteroid use with ablation does not lead to significantly improved outcomes in this population, regardless of follow-up duration.

The meta-analysis of RCTs shows a forest plot evaluating the efficacy of corticosteroids with ablation in preventing AF recurrence, analyzed over three different follow-up durations: less than three days, three to 30 days, and up to three months. For treatments under three days, the OR is 0.56 (95% CI: 0.24 to 1.32), indicating no significant effect with high heterogeneity (I² = 67%). The three- to 30-day group shows an OR of 0.77 (95% CI: 0.40 to 1.50), without significant benefit and lower heterogeneity (I² = 16%). Notably, the subgroup treated for up to three months demonstrates a substantial reduction in recurrence, with an OR of 0.40 (95% CI: 0.21 to 0.77) and low heterogeneity (I² = 19%). When all periods are combined, corticosteroids exhibit a protective effect with an OR of 0.48 (95% CI: 0.29 to 0.78) and moderate heterogeneity (I² = 42%).

These findings suggest a potentially useful effect of corticosteroids on reducing long-term AF recurrence, with benefits becoming significant as the post-treatment period extends to three months. Future research should focus on understanding the optimal duration and dosing of corticosteroid treatment to maximize benefits and minimize risks, especially in the immediate post-treatment period, where the data show less clarity and higher variability. Additionally, further studies should explore the mechanisms through which corticosteroids exert their effects over different durations to tailor treatment protocols better.

In conclusion, all RCTs and cohort studies suggest no significant benefit of corticosteroids in preventing short-term (less than three months) AF recurrence. RCTs indicate that corticosteroid use with ablation significantly reduces the recurrence of AF over three months but such a statistically significant effect is not seen in cohort studies following AF recurrence up to one year. This suggests that the duration and timing of corticosteroid therapy play a critical role in its effectiveness, particularly in preventing recurrences.

Quality-of-study assessment using Grade Pro is shown in Table 7 [48].

**Table 7:** Grade Pro assessment of the study [48]. AF: atrial fibrillation; CI: confidence interval; OR: odds ratio.

Certainty assessment							No of patients		Effect		Certainty
Number of studies	Study design	Risk of bias	Inconsistency	Indirectness	Imprecision	Other considerations	Corticosteroid	Placebo	Relative (95% CI)	Absolute (95% CI)	
AF Recurrence in Observational Studies											
4	Non-randomized studies	Serious	Not serious	Not serious	Very serious	None	148/625 (23.7%)	130/503 (25.8%)	OR 0.85 (0.64 to 1.14)	30 fewer per 1,000 (from 76 fewer to 26 more)	⨁◯◯◯ Very low
AF Recurrence in Randomized Controlled Trials											
4	Randomized trials	Not serious	Not serious	Not serious	Very serious	None	59/397 (14.9%)	114/427 (26.7%)	OR 0.48 (0.29 to 0.78)	118 fewer per 1,000 (from 171 fewer to 46 fewer)	⨁⨁◯◯ Low

Limitations of the Systematic Review and Meta-analysis

The limitations of this study primarily stem from the heterogeneity of the included studies, which employed various designs such as cohort studies, retrospective cohort studies, and RCTs, each with differing follow-up durations and outcome measures. This variability can affect the comparability of results and the generalizability of findings across different patient populations. Additionally, some studies had small sample sizes, potentially limiting the statistical power and robustness of the conclusions drawn. The reliance on various definitions of AF recurrence and varying criteria for corticosteroid treatment further complicate data interpretation. Duration, dose, or type of steroid use with ablation was not taken into account which does not clarify the optimal use of steroid. Moreover, the assessment of bias in the included studies suggests potential issues related to selection bias and confounding factors that were not uniformly controlled for, which could influence the observed outcomes. Finally, the follow-up periods varied among studies, leading to concerns about the long-term applicability of the results and the potential impact of evolving treatment protocols on AF recurrence rates.

## Conclusions

This meta-analysis demonstrates that the impact of corticosteroid therapy on AF recurrence varies depending on the duration of treatment. Observational studies showed no significant effect on AF recurrence within the first three months or extended up to 12 months whether corticosteroids were used with ablation. However, the subgroup analysis of RCTs revealed that the use of corticosteroid with ablation did not significantly reduce AF recurrence in the short term (less than three days) or intermediate (three to 30 days) but was associated with a statistically significant reduction in recurrence rates of AF in three months or beyond. This suggests that using corticosteroids with ablation procedures may be beneficial in reducing long-term AF recurrence. Thus, the findings support considering corticosteroids with ablation procedures to achieve better outcomes in preventing AF recurrence. However, further research is needed to establish the optimal duration and timing of treatment.
